# Lenalidomide in Diffuse Large B-Cell Lymphoma

**DOI:** 10.1155/2012/861060

**Published:** 2012-11-20

**Authors:** Catherine Thieblemont, Marie-Hélène Delfau-Larue, Bertrand Coiffier

**Affiliations:** ^1^Hematology and Oncology Department, Hôpital Saint-Louis, AP-HP and IUH, INSERM U728, University Paris VII, 75010 Paris, France; ^2^Immunbiology Department, Groupe Hospitalier Henri Mondor-Albert Chenevier, AP-HP, 94000 Créteil, France; ^3^Hematology, Centre Hospitalier Lyon Sud, 69230 Pierre-Bénite, France

## Abstract

Diffuse large B-cell lymphoma (DLBCL) is the most common form of non-Hodgkin's lymphoma (NHL) in adults. Even if the natural history of DLBCL has been improved with the advent of immunochemotherapy, the survival results obtained with current treatment options clearly indicate that new agents or novel approaches are needed. Lenalidomide (Revlimid, Celgene Corporation, Summit, NJ, USA), an analogue of thalidomide, is an immunomodulatory drug with pleiotropic mechanisms of action potentially adding to immunochemotherapy. We present here the biological rational for the use of lenalidomide in DLBCL in light of recent advances in the pathophysiology of the disease and the therapeutic results of the most recent trials published in literature or reported in meetings in relapsed/refractory situations as well as in first-line treatment.

## 1. Introduction

The incidence of non-Hodgkin's lymphoma (NHL) has been increasing worldwide during the last 40 years and accounts for 4% of all cancer diagnoses. Among the NHL, diffuse large B-cell lymphoma (DLBCL) is the most common form in adults, accounting for 25–30% of NHL cases [1] and is recognized as an entity since the first classification of NHL [2]. However, complexity and heterogeneity of the disease have been demonstrated over the past ten years, first by the most recent WHO classification including not less than 13 different subentities [3], and second by the biological analyses, particularly the gene expression profiling analyses dividing the disease in at least two molecular subgroups, that is, germinal center B-cell-like (GBC)- and activated B-cell-like (ABC)-DLBCL [4]. These biological analyses have been able not only to capture the molecular heterogeneity of tumor cells [4], but also to demonstrate the existence of a complex interaction between the tumor and its microenvironnement involving multiple signaling pathways and regulatory mechanisms [5].

Standard first-line treatment for DLBCL patients is based since 2002 on the association of rituximab and CHOP (cyclophosphamide, vincristine, doxorubicin, and prednisone) [6]. Even if the natural history of DLBCL has been improved with treatments based on this association, there is clearly a need of improvement of long-term results. With R-CHOP, the expected 5-year and 10-year OS rates are, respectively, 58% and 43.5% [7, 8]. To improve these results, several changes to conventional R-CHOP have emerged either in shortening intervals between cycles [9] or giving alternative regimens with intensified doses of chemotherapy [10]. R-EPOCH (etoposide doxorubicin, vincristine associated with bolus cyclophosphamide, prednisone) has demonstrated to give an OS rate of 73% [11]. In patients <60 years old, GELA has developed R-ACVBP (doxorubicin, cyclophosphamide, vindesine, bleomycin, prednisone) given every 14 days [10] and subsequently demonstrated a superiority of R-ACVBP compared to R-CHOP in several additional randomized studies [12, 13]. However none of these intensified regimens are appropriate for patients with comorbidities or with older age, and the survival results obtained with these current treatment options for patients with DLBCL indicate that new treatment modalities are needed.

## 2. Part I: Biological Relevance of Lenalidomide for the Treatment of DLBCL

**Figure 1:**
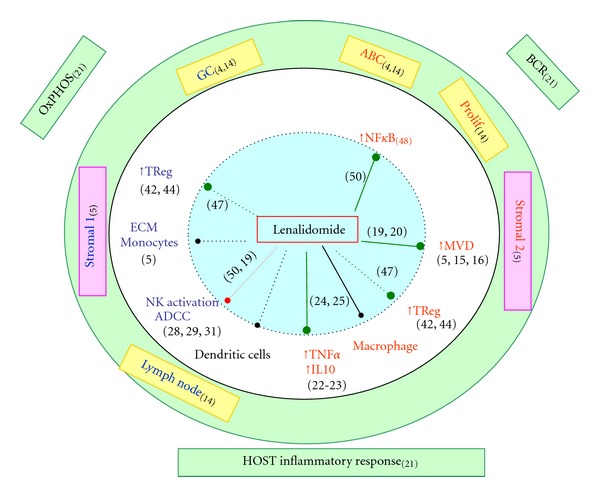
Biological effects of lenalidomide. Colored insets show the main transcriptomic signatures described in DLBCL. Just outside the circle are the signatures with prognostic impact. Inside the circle are indicated the factors studied in DLBCL, either with bad (red characters), good (blue), or undetermined (black) prognostic impact. Arrows indicate a negative (green), positive (red), or undetermined (black) regulation of lenalidomide on those factors in DLBCL. ECM: extracellular matrix components. MVD: microvascular density.

The antitumoral properties of lenalidomide in hematologic area (review in [14]) have been first studied in myeloma, and more recently in myelodysplastic syndromes and lymphomas, and can be grouped in 3 categories: (i) anti-angiogenesis, (ii) immune modulation, and (iii) direct tumor cell toxicities. Some progress on the understanding of DLBCL physiopathology enables us to speculate on biological pathways that could be targeted by lenalidomide (Figure 1).

### 2.1. Antiangiogenic Effects

Beside the two biologically and clinically distinct GC and ABC molecular subtypes of DLBCL defined by a tumoral cell signature [4, 15], different stromal gene signatures have been linked to prognosis [5, 15]. One was associated with reduced survival, includes markers of endothelial cells, regulators of angiogenesis, and was shown to correlate with a quantitative measure of blood-vessel density (MVD) in tumor [5]. Unfavorable prognostic of high MVD has been confirmed on tissue microarray (TMA) in CHOP [16], and R-CHOP [17] treated DLBCL patients. Vascular endothelial growth factor (VEGF)-A is the most prominent proangiogenic factor and value of serum VEGF has prognosis impact in lymphomas (review in [18]). However, the pathogenic association of MVDs and VEGF expression by tumor cell in DLBCL remain controversial [19]. On the basis of these results and on results on *in vivo* model [20], it can be hypothesized that patients with DLBCL characterized by increased tumor MVD may benefit from antiangiogenic effect of lenalidomide [21]. 

### 2.2. Effects on the Immune Microenvironment

#### 2.2.1. Action on Proinflammatory Cytokines and T-cell Activation

Using whole genome arrays, and multiple clustering methods, Monti and colleagues have identified 3 discrete subsets of DLBCL [22], including one characterized by increased expression of T-natural killer cell receptor and activation pathway components, complement cascade members, macrophage/dendritic cell markers, and inflammatory mediators which has been referred as “host response” (HR) signature. Consistent with the signature of an ongoing inflammatory/immune response, HR tumors had increased expression of interferon-induced genes, tumor necrosis family (TNF) ligands and receptors, cytokine receptors, adhesion molecules, and extracellular matrix components. The role of microenvironment associated cytokine in DLBCL physiopathology has been approached in another way. Host immune gene polymorphisms including TNF*α* and IL10 predict late survival in DLBCL patients in the prerituximab era [23]. In accordance, the combined elevation of both TNF*α* and IL-10 in sera of DLBCL patients at diagnosis has been shown to negatively impact on prognosis [24]. The effect of lenalidomide on TNF*α* production depends on immunological context. Lenalidomide has been shown to inhibit the production of proinflammatory cytokines including TNF*α* and to elevate the production of anti-inflammatory cytokine IL-10 from human PBMCs stimulated by LPS. In contrast, a strongly elevated production of TNF*α* [25] by CD3 stimulated T-cell during costimulation by lenalidomide [26] has been reported. In this model, the augmentation of TNF*α* is due to production by both CD4+ and CD8+ T cells, and is dependent on IL2-mediated signalling. Thus, depending on immune environment, lenalidomide could have differential impact on DLBCL tumors.

#### 2.2.2. Action on NK Cells

Under normal circumstances of immune surveillance, human NK cells have inhibitory receptors that recognize MHC class I molecules as their cognate ligands on virtually every cell in the body [27] and activating receptors that sense stressed cells, that is, transformed or infected cells. Thus, NK cells spare healthy cells that express self-MHC class I molecules and low amounts of stress-induced self-molecules, whereas they selectively kill stressed target cells that downregulate MHC class I molecules and/or upregulate stress-induced self-molecules [28]. Investigating spontaneous B-cell lymphoma development in aging *β*2m-deficient mice, Street et al. [29] have shown that NK cells are critical in innate immune surveillance of B-cell lymphomas. In human, alteration of *β*2m expression leading to an altered HLA-I staining patterns has been report in 40/53 (75%) of DLBCL [30], a situation prone to activate NK cells. However, 75% of such HLA-I deficient tumors have concomitant alterations of CD58 (LFA3) expression leading to a potential defect in adhesion and activation of NK cells as it has been shown for LFA1/ICAM1 [31]. The frequency of *β*2m and CD58 expression defect was comparable in GCB and ABC DLBCL subgroups, but the correlation with the HR “Monti classification” has not been reported. We have previously shown that peripheral blood NK cell count deficiency was associated with lower response rate to CHOP like induction regiment [32] in DLBCL suggesting a cooperative contribution of the immune system to the chemotherapeutic response, a feature demonstrated in several mouse model [33]. *In vitro* addition of lenalidomide to PBMC of healthy individuals significantly increased their NK cell natural cytotoxicity (Davies et al., 2001 [34]) in a CD4+T cell and IL-2 dependent manner [35, 36]. The *in vivo* effect of lenalidomide on NK cell of patients with DLBCL treated with lenalidomide is under study.

The introduction of rituximab in therapeutic arsenal has greatly changed the clinical course of DLBCL [6, 37, 38]. Beside natural cytotoxicity mentioned above, NK cell may be involved in Rituximab mediated antidody dependant cytotoxicity (ADCC) by the engagement of the Fc portion of the antibody on their Fc*γ*RIII (CD16) receptors. It has been shown that Fc*γ*RIII polymorphisms impact on antibody binding, resulting in more effective antibody-dependent cellular cytotoxicity *in vitro* [39]. The association with Fc*γ*RIII polymorphisms and clinical outcome has been used to argue for an ADCC mechanism of action of rituximab *in vivo*, but the association is less significant in DLBCL [40] than initially report in follicular lymphoma [41].

 Lenalidomide has been shown to enhance the NK-cell-mediated ADCC and NK cell IFN-*γ* production in a series of functional *in vitro* experiments using rituximab coated NHL cell lines, including one cell line derived from a DLBCL patient [42].

#### 2.2.3. Action on Regulatory T Cells

Regulatory T cells (T_REG_), defined as CD4^+^CD25^+^ T cells, play an important role in the immune system, not only by inhibiting autoimmunity, but also by hampering the antitumour response [43]. In human NHL (including 6 DLBCL) it has been shown *in vitro*, that intratumoral CD4^+^CD25^+^ cells can inhibit the proliferation of activated anti-tumour CTLs, and can inhibit the proliferation and the secretion of IFN*γ* and IL-4 by infiltrating CD4+CD25-T cells [44]. The expression of FOXP3 has been evaluated by immunohistochemical study on paraffin-embedded DLBCL tumor specimens and the number of FOXP3+ regulatory T cells has been first shown to be not predictive of clinical outcome [45]. However, the correlation between FOXP3+ infiltrating T cells and prognosis has been subsequently evaluated independently in GC and non-GC DLBCL subgroups [46], as defined by Hans algorithm [47]. Despite the fact that the absolute FOXP3+ cell numbers were similar in GC and non-GC DLBCL, a high amount of tumor-infiltrating FOXP3+ cells was of good prognostic value (DFS) in GC but was associated with an adverse clinical outcome in non-GC subgroup. In this study, localization of FOXP3+ cells within tumor, a feature that has been shown to impact their clinical value in solid tumor [48], has not been evaluated.

Lenalidomide can inhibit the proliferation of FOXP3+ CTLA4+CD4+CD25^high^ T_REG_ cells in healthy donor PBMCs cultured for 7days with IL-2 [49]. Moreover, lenalidomide inhibit the suppressor function of the T_REG_ cells against autologous responder cells *in vitro*. This inhibitory activity is associated with reduction of FOXP3 and OX40 expression. However, to our knowledge, nothing has been report on the effect of lenalidomide on T_REG_ extracted from DLBCL tumor samples. 

### 2.3. Direct Effect on Tumor Cells

A hallmark of ABC DLBCL is the constitutive activation of the NF*κ*B pathway, on which they rely for survival and proliferation [34]. NF*κ*B activation in mediated through oncogenic driver mutations affecting B-cell receptor-NF*κ*B signaling (review in [50]). Beside effects on microenvironment, lenalidomide has been shown to have a direct effect on DLBCL cell lines, with a decrease in NF*κ*B activity and an arrest in DNA synthesis [51].

## 3. Part II: Lenalidomide and Treatment of DLBCL

### 3.1. Response to Lenalidomide in Relapsed/Refractory DLBCL

Data emerging from early clinical trials demonstrated that lenalidomide has a significant activity against relapsed/refractory DLBCL either as monotherapy or as an association with rituximab. Published results are shown in Table 1.

The first phase II trial was a single-arm, multicenter trial (NHL002) that evaluates the safety and efficacy of lenalidomide oral monotherapy (25 mg/day during 21 days every 28 days) in 49 patients with relapsed or refractory aggressive NHL [52]. Among them, 26 patients presented a relapsed/refractory DLBCL. The median age was 65 years. All these patients were heavily pretreated with a median of four prior treatment regimens. Overall response rate (ORR) was 19%  (*n* = 5/26), including 3 complete responses (CRU + CR) and 2 partial responses (PR).

An international phase II study (NHL003) was then conducted enrolling 218 patients with refractory/relapsed B-cell aggressive lymphoma, and confirmed the efficacy of lenalidomide in this category of patients [53]. One hundred and eight patients with diffuse large B-cell lymphoma were included. As the previous study, the treatment consisted in lenalidomide 25 mg orally once daily on days 1–21 of every 28 day cycle. Thirty patients (28%) exhibited an objective response (8 CR, and 22 PR). Interestingly, response to lenalidomide therapy was independent of the tumor burden, and of the number and the type of prior treatment. Compared to other type of lymphomas included (mantle cell lymphoma, transformed large B-cell lymphoma, follicular lymphoma, grade III), progression-free survival (PFS) of the patients with DLBCL was the shortest.

In contrast, patients with large cell NHL of the transformed type (*n* = 33) had substantially better results [53]. Median PFS was of 5.1 months and median response duration of 12.8 months. These results were further explored in a study analysing 33 patients with transformed follicular lymphoma (tFL), transformed chronic lymphocytic leukaemia/small lymphocytic lymphoma (tCLL/SLL) [55]. Lenalidomide was administered at the same dosage. Among patients with tFL, ORR was 57%, with a median response duration of 12.8 months. None of the patients with tCLL/SLL responded to lenalidomide monotherapy.

These encouraging results are confirmed in a retrospective study (REVEAL study) showing an objective response rate after 3 cycles at 69.2% in heavily pretreated patients with relapsed LNH [59].

When associated to rituximab, results of ORR seems equivalent to lenalidomide alone, with an ORR of 35% [54]. However number of CR seems higher as almost all but one responding patients were in CR. In this trial, the treatment plan comprised an induction phase with lenalidomide (20 mg/day, D1-D21 of a 28-day cycle for 4 cycles) and rituximab (375 mg/m2 on day 1 and day 21 of each cycle—total of 4 cycles) and maintenance therapy proposed to the responders (CR, PR SD) with lenalidomide. Interestingly one patient in PR after induction converted to CR during the maintenance.

Interestingly maintenance with lenalidomide is currently tested in patients with relapsed DLBCL who achieved at least a partial response to second-line chemotherapy (ICE, DHAP/DHAOx, or MINE regimen) and rituximab at the dose of 25 mg once daily for 21 days out of 28 until progression (NCT00799513).

### 3.2. Toxicity of Lenalidomide Alone in DLBCL

When lenalidomide is used in monotherapy at the “standard” dose of 25 mg/d D1–D21 cycling at 28 days, the most common grade 3 and 4 adverse events are neutropenia occurring in 33% to 41% of the patients, and thrombocytopenia in 20% of the patients. The neutropenia was rarely complicated with a febrile neutropenia, reported in 2–6% of the patients. No neuropathy was reported. Deep vein thrombosis was described in 2% of the patients. Other grade III-IV toxicities were anemia (<10%) and asthenia in 5% of the patients. These toxicities required a dose reduction in one third of the patients in both trials [52, 53] (Table 2). The median time to first dose reduction or interruption of treatment was 33 days [53]. The most common reasons for dose reduction were neutropenia (56%) and thrombocytopenia (31%) [53].

### 3.3. Lenalidomide in First-Line Treatment in DLBCL

Combination of lenalidomide and standard R-CHOP21 have been recently published or reported in meetings by several groups in phase I-II [56]. This strategy of “R2-CHOP” was proposed to patients in first-line treatment (Table 3). The lenalidomide dose levels tested were between 5 mg up to 25 mg/day. Duration of treatment by cycle was between 10 days to 14 days. Dose limiting toxicity principally occurred because of haematological toxicity described as the most frequent adverse event. Grade III-IV neutropenia occurred in 28% to 88% of the patients, and Grade III-IV thrombocytopenia in 10 to 30% of the patients. Grade I-II neuropathy was observed in half of the patients, and grade III-IV in around 10% of the patients.

Beside concomitant association of lenalidomide and R-chemotherapy, alternative way to administer lenalidomide in front-line is a strategy of maintenance therapy after an induction of R-CHOP. Lenalidomide seems attractive to test in maintenance with several positive arguments. It is an oral drug, easy to administer. The early antitumoral efficacy and immunomodulatory effect have been shown, and finally tolerance is acceptable. This strategy is currently investigated in an international trial conducted by the LYSA (EUDRACT Number: 2008-008202-52), where lenalidomide is proposed in maintenance after R-CHOP21 or R-CHOP14 in responding patients (CR + PR) [60] aged from 60 to 80 years old with at least one adverse IPI prognostic factors. 

### 3.4. Cell of Origin and Response to Lenalidomide

Based on the biological rational of Lenalidomide and the new categorization of DLBCL [4], Hernandez-Ilizaliturri et al., recently reported the response to lenalidomide in relapsed/refractory DLBCL in analyzing them within their subgroups: germinal center B-cell (GCB-)-like- or nongerminal center B-cell (non-GCB-)like- DLBCL [61]. Forty patients were retrospectively analyzed using the Hans's algorithm based on the expression of CD10, BCL6, and IRF4/MUM1 by immunohistochemistry [47]. Twenty-three were classified as GCB-like DLBCL and 17 as non-GCB-like DLBCL. Differences were observed in responses rates, PFS and OS. ORR rate was significantly higher in patients with non-GCB-like DLBCL compared to patients with GCB-like DLBCL (OOR rates, 53% versus 9%, *P* = .006). Complete response rate was 23.5 versus 4.3%. Median progression-free survival was 6.2 months versus 1.7 months. No difference in OS was yet observed.

## 4. Conclusion

Lenalidomide is a promising drug in DLBCL in relapse as well as in front-line therapy. Several trials have reported interesting results in monotherapy as well as in association with rituximab alone or with immunochemotherapy. Tolerance seems acceptable and long term results of the recently published trials should in the future help to define the place of this drug in the therapeutic strategy of patients with DLBCL. Numerous new therapeutic molecules are under development or in phase I/II evaluation and some additional biological works are necessary to decipher the precise mechanism of action of lenalidomide in DLBCL subgroups in order to develop rational combinations [62].

## Figures and Tables

**Table 1:** Response to Lenalidomide in relapsed/refractory diffuse large B-cell lymphomas.

Lenalidomide	Monotherapy		Association	
Name of the protocol	NHL002 [52]	NHL003 [53]	Lenalidomide and rituximab [54]	
Year of publication	2008	2011	2011	
Type of study	Multicentric	International	Multicentric	
Phase	Phase II	Phase II	Phase II	
Treatment	Lenalidomide	Lenalidomide	Lenalidomide and rituximab	
Dose of lenalidomide:	25 mg/d, D1–21	25 mg/d, D1–21	20 mg/d, D1–21	
	every 28 days	every 28 days	every 28 days	
Duration or treatment or No. of cycles	52 weeks	—	4 cycles + maintenance (*n* = 10 pts)	
No. of patients	49	267	23	
No. of DLBCL	26	108	23	
Response*			Induction	Complete therapy
ORR *n*, (%)	5 (19)	30 (28)	8 (35)	8 (35)
CR *n*, (%)	1 (3)	8 (7)	7 (31)	8 (35)
CRU *n*, (%)	2 (8)	—	—	
PR *n*, (%)	2 (8)	22 (20)	1 (4)	0 (0)
Stable disease *n*, (%)	7 (27)	23 (21)	2 (8)	2 (8)
Progression *n*, (%)	14 (54)	40 (37)	13 (57)	
Followup		9.2	16	
Median time to response (month)	PR: 1.9 (1.2–3.7) CR: 4.3 (1.9–10.5)	1.9 (1.4–11.5)	—	
Median response duration (month)	6.2 (0–12.8)	1.6	—	
PFS, Median (month)	4	2.7	1-year DFS 34.8%	

ORR: overall response rate, CR: complete response, CRU: complete response unconfirmed, PR: partial response, PFS: progression free survival.

NHL002: the results of response are specifically reported for DLBCL.

NHL003: the results of response are specifically reported for DLBCL.

**Table 2:** Grade III-IV toxicities with lenalidomide as monotherapy in relapsed/refractory DLBCL.

	NHL002 [52]	NHL003 [53]
	%	%
Neutropenia	33	41
Febrile neutropenia	6.1	2.3
Thrombocytopenia	20.4	18.4
Anemia	6.1	9.2
Fatigue	6.1	4.6
Deep vein thrombosis	2	2.3
Neuropathy	0	0

**Table 3:** Response to lenalidomide in patients with diffuse large B-cell lymphoma in first-line treatment.

Name of the protocol	R2-CHOP [56]	LR-CHOP21 [57]	R2-CHOP [58]	
Year of publication	2011	2010	2011	

Type of study	Monocentric	Multicentric-IIL	Multicentric	
Phase	Phase I	Phase I-II	Phase I-II	
Treatment	Lenalidomide and R-CHOP21	Lenalidomide and R-CHOP21	LenalidomideandR-CHOP21	
Dose of lenalidomide:	15 to 25 mg/d, D1–10	5 to 20 mg/d, D1–14	5 to 25 mg/d, D1–14	
	every 21 days	every 21 days	every 21 days	
No. of cycles	6	6	6	
No. of patients with DLBCL	24	21	27	
Recommended dose in function of DLT	25 mg	15 mg	25 mg	
Toxicity		
Hematologic	Grade III-IV	Grade III-IV	Grade III-IV	
Anemia	21%	4%	—	
Neutropenia	88%	28%	59%	
Thrombocytopenia	29%	10%	30%	
	Grade III	Grade III	Grade I-II	Grade III
Peripheral neurotoxicity	8%	14%	48%	0%
Vascular thrombosis	8%		7%	
Response		
ORR *n*, (%)	22 (87.5)	16 (72)	27 (100)	
CR *n*, (%)	18 (77)	15 (71)	20 (74)	
PR *n*, (%)		1 (5)	7 (26)	
Stable disease *n*, (%)			—	
Progression *n*, (%)	5 (21)	5 (16)	—	

R-CHOP: rituximab 375 mg/m^2^ D1, cyclophosphamide 750 mg/m^2^ D1, doxorubicin 50 mg/m^2^ D1, vincristine 1.4 mg/m^2^ D1 (capped at 2.0 mg) prednisone 50 mg/m^2^ D1–5.

DTL: dose limiting toxicity.
